# Full-lactation performance of multiparous dairy cows with differing residual feed intake

**DOI:** 10.1371/journal.pone.0273420

**Published:** 2022-08-26

**Authors:** Johanna Karlsson, Rebecca Danielsson, Maria Åkerlind, Kjell Holtenius

**Affiliations:** 1 Department of Animal Nutrition and Management, Swedish University of Agricultural Sciences (SLU), Uppsala, Sweden; 2 Växa Sverige, Uppsala, Sweden; Tokat Gaziosmanpasa Universitesi, TURKEY

## Abstract

Residual feed intake (RFI) is an efficiency trait underpinning profitability and environmental sustainability in dairy production. This study compared performance during a complete lactation of 36 multiparous dairy cows divided into three equal-sized groups with high (HRFI), intermediate (IRFI) or low RFI (LRFI). Residual feed intake was determined by two different equations. Residual feed intake according to the NorFor system was calculated as (RFI_NorFor_) = (NE_intake_)–(NE_maintenance_ + NE_gestation_ + NE_milk_—NE_mobilisation_ + NE_deposition_). Residual feed intake according to the USA National Research Council (NRC) (RFI_NRC_) was calculated as: RFI = DMI − predicted DMI where predicted_s_ DMI = [(0.372× ECM)+(0.0968×BW^0.75^)]×(1−e^−0.192×(DIM/7+3.67)^). Cows in the HRFI_NorFor_ group showed higher daily CH_4_ production, CH_4_/ECM and CH_4_ yield (g/kg DMI) than IRFI_NorFor_ and LRFI_NorFor_ cows. Cows characterized by high efficiency (LRFI_NorFor_) according to the NorFor system had lower body weight. Dry matter intake and apparent dry matter digestibility were not affected by efficiency group but milk yield was lower in the low efficiency, HRFI_NorFor,_ group. Cows characterized by high efficiency according to the NRC system (LRFI_NRC_) had lower dry matter intake while yield of CH_4_ was higher. Daily CH_4_ production and CH_4_ g/kg ECM did not differ between RFI_NRC_ groups. Dairy cows characterized by high efficiency (both LRFI_NorFor_ and LRFI_NRC_ cows) over a complete lactation mobilized more of their body reserves in early lactation as well as during the complete lactation. The results also indicated great phenotypic variation in RFI between different stages the lactation.

## Introduction

The efficiency of dairy cows, as defined as the fraction of feed energy captured in milk, has been increased through genetic selection, nutrition, and management. Reduced maintenance requirement through increased yield of milk has been the overwhelming driver of enhanced efficiency. However, efficiency can also be improved without reducing the maintenance requirement. Efficiency in that case can be estimated using residual feed intake (RFI), expressed as the difference between actual feed intake of an individual and that expected based on its energy requirements, and is not related to level of production [1]. At present, RFI is commonly determined as the deviation of actual dry matter intake (DMI) or energy intake of a cow from the average intake of other cows fed and managed in the same cohort, after adjusting for three major energy sinks: body weight (related to maintenance), milk energy output, and body energy change [2]. Animals with negative RFI values are more efficient, because they eat less for a given production level or produce more for a given intake level [3]. Estimation of RFI in lactating dairy cattle is particularly difficult due to fluctuations in the energy balance (EB) throughout the lactation cycle. Phenotypic correlations between RFI estimates made for sub-periods of the full lactation (early, mid-, and late lactation) are reported to be poor [4, 5]. Correlation analysis between full lactations and different sub-periods is reported to yield stronger relationships, with a 64–70 day test period between 150 and 220 days in milk (DIM) giving reliable estimates [6]. Negative energy balance (NEB) in early lactation is strongly correlated with RFI and it was suggested that on a given time period animals who are classified as more feed efficient are more likely to have a lower energy balance [7]. The interactions between NEB, fertility, and metabolic diseases are well-established [8, 9]. This raises concerns that improving feed efficiency using RFI might result in increased loss of body condition score (BCS) and deeper NEB in early lactation, impairing health and fertility in dairy cows [7, 10, 11]. Approximately 30% of dietary energy is lost by cycling through body reserves, representing a major inefficiency [7]. Thus, cows with marked fluctuations in EB throughout the lactation cycle are less efficient. The major components affecting RFI relate to alterations in conversion of gross energy (GE) to net energy (NE) [2]. Conversion of GE to NE can be divided into digestive and metabolic efficiency. In beef cattle, digestive efficiency seems to be weakly associated with RFI [12]. It is well known that enteric methane emissions from cattle and other ruminants reduce the efficiency of converting GE to NE. High feed efficiency could potentially lead to a decline in enteric methane emissions, due to the positive phenotypic correlation between methane emissions and RFI [13].

The aims of this study were to characterize and compare traits in cows with high, intermediate or low RFI, estimated by the equations NorFor [14] and the USA National Research Council [15] respectively to examine phenotypic correlations between RFI for the full lactation and shorter sub-periods.

## Material and methods

### Animals, experimental design, and housing

The Uppsala Ethics Committee, Sweden approved all handling of animals for Animal Research, (diary number C 99/16). In total, 36 cows (13 Swedish Holstein (SH) and 23 Swedish Red (SR) breed) were studied over one full lactation period until dry off. The cows were all multiparous (19 in second lactation, 17 older (third to seventh lactation)).

The cows were housed in a loose house with rubber mats and sawdust-bedded cubicles. They were milked voluntarily in a single-station automatic milking system (VMS, DeLaval International AB, Tumba, Sweden) with the FeedFirst cow traffic system, which resulted in 2.6 milkings per day (SD = 0.4). Milking interval was set to 6 h for cows with low somatic cell count (SCC) and 4 h for cows with high SCC (>100 000 cells/mL), both with a maximum of 12 h between milkings.

### Diets and feeding

Chemical composition of silage and concentrates is shown in Table 1. All cows had free access to grass-clover silage. The concentrates were pelleted and fed individually in concentrate dispensers (FSC400, DeLaval International AB, Tumba, Sweden) and in the milking unit.

**Table 1:** Chemical composition (mean ± SD) of experimental feeds (g/kg DM, unless otherwise stated). Where standard deviation is reported, the number of samples used for analyses of chemical composition was n = 31 for silage and n = 32 for concentrates (except fat content, where n = 5).

Variable	Grass-clover silage	Byproduct-based concentrate
DM, g/kg	407 ± 50	872 ± 8.4
Ash	86.4 ± 4.0	65.4 ± 4.8
Crude protein	166 ± 17	151 ± 6.1
Crude fat	-^1^	47.8 ± 6.1
NDF^2^	425 ± 35	361 ± 10
Starch	-^1^	54.4 ± 11
WSC^3^	2.0 ± 1.4	5.3 ± 1.7
NE_L_, MJ/kg DM	6.54 ± 0.06^4^	6.59^4^

^1^Not analyzed.

^2^Neutral detergent fiber.

^3^Water-soluble carbohydrates.

^4^Calculated in NorFor (Åkerlind and Volden, 2011) based on chemical composition, and tabulated values and estimates for digestibility characteristics where analytical data were lacking.

The cows were also subjected to another study [16] and therefore fed two different levels of concentrate. The concentrate ration was increased over 21 d, starting at 3 kg/d at calving, to a maximal concentrate ration of 12 kg/d or 6 kg/d in the two different groups which included concentrate offered in the milking unit. The cows stayed on that rations until 210 DIM, when the concentrate amount was gradually decreased to 0 kg/d over 95 d.

All cows had access to a small grass-covered permanent paddock for exercise and recreation at night-time between mid-May to mid-August, in compliance with Swedish animal welfare law. Individual pasture intake, estimated to be 0.5 kg DM/d, was not included in total DMI.

### Measurements and sample collection

Individual daily forage intake was recorded automatically by 20 forage troughs on weight scales (CRFI, BioControl Norway A/S, Rakkestad, Norway). Daily concentrate intake was recorded by dispensers (FSC400, DeLaval International AB, Tumba, Sweden). The equipment used for forage intake recording was calibrated weekly and the dispensers used for concentrate intake recording were calibrated monthly. The raw data on individual daily forage intake showed improbably high feed intake for some cows and days, caused by some cows throwing silage out of the forage troughs. The rate of feed intake appears to be related to DMI, with little individual variation [17]. Therefore intake for feeding occasions with intake rate >8.28 g/s of fresh weight (95% confidence level of all eating occasions for all cows included in the study) was replaced with individual intake estimates derived from daily average intake rate <8.28 g/s. Forage DMI and total DMI were treated as missing values for days when total DMI divided by metabolic body weight (BW) was above 0.22 kg/kg (95% confidence level). The cows were automatically weighed every time they passed through a sorting gate when leaving the feeding area, and mean daily BW was recorded (AWS100, DeLaval International AB, Tumba Sweden). Body condition score (scale 1–5) was assessed automatically with a 3D camera (DeLaval International AB, Tumba, Sweden) every time the cows left the milking station. Weekly mean BW and BCS were calculated from daily mean BW and BCS, respectively.

Silage was sampled five times a week and pooled into three-week periods for analysis of chemical composition, while concentrates were sampled once a week and pooled into four-week periods for analysis. Silage samples were collected in plastic bags and stored at -20°C until analysis, while concentrate samples were stored at room temperature in plastic bags. Spot samples of feces for estimation of digestibility were collected on three consecutive days in early (23±5.5 DIM) and mid- lactation (134±6.4 DIM). The feces samples were stored at—20°C until further processing.

Milk sampling for milk composition analysis was carried out every second week until the cows were dried off, when milk samples were taken at two consecutive milkings. The milk meter (MM25, DeLaval International AB, Tumba, Sweden) used for measuring milk yield and the milk sampler (DeLaval Milk Sampler, DeLaval International AB, Tumba, Sweden) have been certified by the International Committee for Animal Recording (Rome, Italy). Milk samples were preserved with bronopol, stored at 8°C, and analyzed within 3 d.

In lactation weeks 2, 4, and 6, blood samples were drawn from the coccygeal vein or artery of the tail-head into 10-mL vacuum tubes containing lithium heparin as anticoagulant (BD Vacutainer, Becton, Dickinson and Company, Franklin Lakes, NJ). The blood samples were centrifuged immediately (4000 rcf, 10 minutes, +4°C) and the blood plasma was transferred to Eppendorf tubes and stored at -20°C until analysis.

### Chemical analysis and calculations

Analyses of feed, milk composition, feces, and blood plasma were performed in the laboratory at the Department of Animal Nutrition and Management, Swedish University of Agricultural Sciences (SLU), Uppsala, Sweden, unless otherwise stated. The DM content of silage was determined by first drying at 60°C overnight, milling, and then drying at 60°C overnight, according to Åkerlind et al. [18]. The DM content of concentrate feeds was determined by drying at 103°C overnight (EC No 152/2009). Ash content in all feeds was determined by ignition at 550°C for 3 h (EC No. 152/2009). Acid-insoluble ash (AIA) content in all feeds was analyzed according to Van Keulen and Young [19]. Feeds were analyzed for crude protein (CP) in an automated Kjeldahl procedure (Foss, Hillerød, Denmark). Ether extract analyses were performed by Eurofins Food & Feed Testing Sweden AB, Jönköping, Sweden, according to EC (EC No. 152/2009). Concentrate samples were analyzed enzymatically for starch (including maltodextrin) according to Larsson and Bengtsson [20]. All feeds were analyzed for neutral detergent fiber (NDF) according to Chai and Udén [21]. Silage was analyzed for water-soluble carbohydrates according to Larsson and Bengtsson [20]. Silage samples were pressed and the silage juice was analyzed for pH.

Net energy content in the feed and energy intake were estimated according to the Nordic feed evaluation (NorFor) system [14] (FST equation revision 1.98 and FRC equation revision 1.90).

Feces samples were freeze-dried, milled, and analyzed for DM, ash, and AIA. The total amount of feces was calculated from the total intake of AIA and the content of AIA in the feces [19]. Total tract apparent dry matter digestibility (DMD) was calculated from intake and excretion of dry matter from feed and feces, as (DM_intake_—DM_feces_)/DM_intake_. The calculation was based on the feces samples taken once daily on three consecutive days and intake data from these three sampling days and the previous day.

Milk samples were analyzed for composition of fat, C18:1 cis-9, protein, and lactose by infrared Fourier transform spectroscopy (CombiScope FTIR 300 HP, Delta Instruments B.V., Drachten, the Netherlands). Lactose was corrected for lactase monohydrate by division by 1.053. Energy-corrected milk (ECM) was calculated based on fat, protein, and lactose concentration according to Sjaunja et al. [22]. Since the cows were milked at different milking intervals in an automated milking system, daily estimates of ECM, milk component yields, and milk composition values were adjusted based on time since last milking.

Residual feed intake according to the NorFor system was calculated as (RFI_NorFor_) = (NE_intake_)–(NE_maintenance_ + NE_gestation_ + NE_milk_—NE_mobilisation_ + NE_deposition_), where each parameter was calculated according to the NorFor system [14] and NE_mobilisation_ and NE_deposition_ were determined based on changes in BCS assessed with the 3D camera. A daily reduction in BCS of 0.01 units contributed 15 MJ NEL/d, while a daily gain in BCS of 0.01 units required 18.5 MJ NEL/d [15]. Residual feed intake according to the USA National Research Council (NRC) (RFI_NRC_) was calculated as: RFI = DMI − predicted DMI [23], where predicted DMI (according to the NRC (2001) equation) = [(0.372× ECM)+(0.0968×BW^0.75^)]×(1−e^−0.192×(DIM/7+3.67)^). At the end of the experiment, the experimental cows (n = 36) were divided into three equal-sized groups, with low, intermediate or high RFI based on RFI_NorFor_ value (LRFI_NorFor_, IRFI_NorFor_ and HRFI_NorFor_), and were also divided into three equal-sized groups based on the RFI_NRC_ value (LRFI_NRC_. IRFI_NRC,_ and HRFI_NRC_). Thus, two separate datasets were created, based on RFI_NorFor_ and RFI_NRC_, respectively.

The persistency of lactation was calculated as average ECM yield during wk 31–40 of lactation divided by average ECM yield during wk 1–10.

Blood plasma was analyzed for metabolites and hormones. Glucose concentration was analyzed enzymatically (D-Glucose UV-method, R-biopharm AG, Darmstadt, Germany). Insulin concentration was analyzed using an enzyme immunoassay method adapted for bovines (Mercodia Bovine Insulin ELISA, Mercodia AB, Uppsala, Sweden), and the concentration of non-esterified fatty acids (NEFA) using an enzymatic colorimetric method (NEFA-HR, FujifilmWako Diagnostics U.S.A. Corporation, CA). The concentration of β-hydroxybutyrate (BHB) in plasma was analyzed with a colorimetric test (MAK041, Sigma-Aldrich, St. Louis, MO).

Methane (CH_4_) emissions were measured by a spot sampling technique where average CH_4_ daily emissions are based on the analysis of multiple short-term spot-samples of air emitted from individual cows. The method used in this study was the infra-red (IR) sniffer method described by Garnsworthy et al. [23], with a similar set-up for measurement and correction for dilution of ambient air as previously described in Danielsson et al. [24]. In brief, a CH_4_ analyzer (Guardian Plus; Edinburgh Instruments Ltd., Livingston, UK) was calibrated using standard mixtures of CH_4_ in nitrogen. The analyzer was attached to the automatic milking system (AMS) and the sampling tube was attached to the concentrate trough within the AMS. The CH_4_ concentrations in air were then measured continuously. Eructation values (peak area and frequency) were used to calculate individual daily mean CH_4_ emission rate during milking. The CH_4_ concentration was logged every second on a datalogger (Simex SRD-99; Simex Sp. z o.o., Gdansk, Poland) and then visualized using logging software (Loggy Soft; Simex Sp. z o.o.). Times of entry to the milking station and cow ID were recognized using the VMS management program (DelPro software, version 3.7; DeLaval International AB), and were coupled with corresponding CH_4_ values from the logger. On average, milking data were recorded 2.6 times per day for each cow, as previously mentioned, but not all recordings were used for CH_4_ calculations, since peaks with height <200 mg/kg above baseline were discarded. Milking occasions with fewer than three recorded peaks were removed from the analysis. On average, 2.2 readings (4.4 peaks) per animal and day were used.

### Statistical analyses

Comparisons between the three RFI groups (low, intermediate and high) with 12 cows in each group, with respect to plasma glucose, insulin, NEFA, BHB, milk C18:1 cis 9, ECM, DMI, DMD, ECM/DMI, BCS, BCS weekly change, BW and BW weekly change were analyzed using PROC MIXED in SAS software (version 9.4, SAS Institute Inc., Cary, NC):

Y_ijklm_ = μ + P_i_ + B_j_ + G_k_ + L_l_ + W_m_ + PW_im_ + BW_jm_ + GW_km_ + ε_ijklm_, where Y_ijklm_ is the dependent variable, μ is the overall mean, P_i_ is the effect of parity i, B_j_ is the effect of breed j, G_k_ is the effect of RFI group k, L_l_ is the effect of concentrate level l, W_m_ is the effect of lactation week m, PW_im_ is the parity × lactation week interaction effect of parity i and lactation week m, BW_im_ is the breed × lactation week interaction effect of breed i and lactation week m, GW_km_ is the RFI group × lactation week interaction effect of RFI group k and lactation week m, and ε_ijklm_ is the random error. The error term in the model was modelled with an autoregressive structure, as observations were made over several lactation weeks for each cow. For CH_4_ related parameters, the interaction of parity × lactation week was removed.

Comparisons between the three RFI groups were made based on one value per cow for persistency of lactation, ΔBW lactation week 14–1, ΔBCS lactation week 14–1. These were analyzed by PROC GLM in SAS software with the following model:

Y_ijkl_ = μ + P_i_ + B_j_ + G_k_ + L_l_ + ε_ijkl_, where Y_ijkl_ is the dependent variable, μ is the overall mean, P_i_ is the effect of parity i, B_j_ is the effect of breed j, G_k_ is the effect of RFI group k, Ll is the effect of concentrate level l, and ε_ijkl_ is the random error.

Several models were tested to combine and account for interactions between variables. The models with the lowest Akaike information criterion were used. All residuals were tested for normality and log-transformation was applied to variables that did not follow a normal distribution. Values presented in the text and tables are least squares means calculated using the LSMEANS/ PDIFF option. Statistically significant differences were determined following Tukey’s adjustment declared at P ≤ 0.05, with trends noted at P ≤ 0.10.

Phenotypic Pearson correlations between RFI_Full-Lact_ (week 1–42) RFI_Early-Lact_ (week 1–14), RFI_Mid-Lact_ (week 15–28), and RFI_Late-Lact_ (week 29–42) were calculated using Minitab 18.1 (Minitab Inc.).

### Results

The mean RFI_NorFor_ (NE/d) of LRFI_NorFor_ cows was negative, of IRFI_NorFor_ close to zero, and that of HRFI_NorFor_ cows was positive (Table 4). The mean RFI_NRC_ (kg DM/d) of LRFI_NRC_ and IRFI_NRC_ cows was slightly negative while that of HRFI_NRC_ cows was close to zero (Table 5).

Table 2 shows the effects of RFI_NorFor_ group during the early stage of lactation. Cows of SH breed had higher insulin values (0.10 μg/L; antilog) compared to SR cows (0.06 μg/L; antilog) (P-value 0.05). No other parameter in Table 2 had any breed effect. The plasma concentrations of glucose and BHB sampled in lactation week 2, 4, and 6 were not affected by RFI_NorFor_ group (high, intermediate, low). However, insulin was lower in LRFI_NorFor_ compared with IRFI_NorFor_ and HRFI_NorFor_ and NEFA was higher in LRFI_NorFor_ compared with HRFI_NorFor_. During the first 14 weeks of lactation, the percentage of C18:1 cis 9 in milk, reflecting mobilization of adipose tissue, was not affected by RFI_NorFor_ group. Cows in HRFI_NorFor_ lost less BCS during the first 14 weeks of lactation compared to the other two groups. The extent of loss of BW was not influenced by RFI group.

**Table 2:** Metabolic and related variables (mean and SEM) in cows in early lactation divided into three groups, with low residual feed intake (LRFI; n = 12), intermediate (IRFI; n = 12) or high RFI (HRFI; n = 12) according to the NorFor (2011) equation. The plasma variables were sampled in lactation week 2, 4, and 6.

*NorFor*								
Variable:	Obs.	LRFI	SEM	IRFI	SEM	HRFI	SEM	P-value
Plasma glucose (mmol/L)	104	2.98	0.11	2.71	0.13	3.07	0.12	0.12
Plasma insulin (log10)	104	-1.309^b^	0.086	-1.02^a^	0.096	-1.05^a^	0.091	0.05
Plasma insulin (μg/L; antilog)	-	0.05	-	0.10	-	0.09	-	-
Plasma NEFA^1^ (log10)	102	-0.30^a^	0.041	-0.38^ab^	0.047	-0.499^b^	0.044	0.019
Plasma NEFA^1^ (mmol/L; antilog)	-	0.50	-	0.42	-	0.32	-	-
Plasma BHB^2^ (log10)	104	-0.01	0.047	0.12	0.053	-0.02	0.050	0.11
Plasma BHB^2^ (mmol/L; antilog)	-	0.98	-	1.32	-	0.95	-	-
Milk C18:1 cis 9 wk 1–14 (% of milk)	220	0.90	0.036	0.97	0.041	0.86	0.039	0.17
ΔBCS^3^ wk 14—wk 1 (scale 1–5)	36	-0.59^b^	0.095	-0.5599^b^	0.117	-0.22^a^	0.109	0.03
ΔBW^4^ wk 14—wk 1 (kg)	36	-37.5	12.62	-40.5	15.55	-16.0	14.49	0.42

^1^Non-esterified fatty acids.

^2^β-hydroxybutyrate.

^3^Body condition score.

^4^Body weight.

^a–b^Means within rows with different superscripts differ significantly (P < 0.05).

Table 3 shows the effects of RFI_NRC_ group during the early stage of lactation. Here there were no effects of breed on any parameter. The plasma concentrations of glucose, insulin and BHB sampled in lactation week 2, 4, and 6 were not affected by RFI_NRC_ group, but NEFA was lower in cows in the HRFI_NRC_ group. During the first 14 weeks of lactation, the percentage of C18:1 cis 9 in milk was lower in the HRFI_NRC_ group, indicating a lower level of adipose tissue mobilization. The HRFI_NRC_ group also lost less BCS compared with IRFI_NRC_ and LRFI_NRC_.

**Table 3:** Metabolic and related variables (mean and SEM) in cows in early lactation divided into three groups, with low residual feed intake (LRFI; n = 12), intermediate (IRFI; n = 12) or high RFI (HRFI; n = 12) according the NRC (2011) equation. The plasma variables sampled in lactation week 2, 4, and 6.

*NRC*								
Variable:	Obs.	LRFI	SEM	IRFI	SEM	HRFI	SEM	P-value
Plasma glucose (mmol/L)	104	2.88	0.124	2.89	0.125	3.01	0.118	0.71
Plasma insulin (log10)	104	-1.21	0.092	-1.21	0.092	-0.99	0.087	0.14
Plasma insulin (μg/L; antilog)	-	0.062	-	0.062	-	0.102	-	-
Plasma NEFA^1^ (log10)	102	-0.40^ab^	0.045	-0.30^a^	0.045	-0.46^b^	0.042	0.03
Plasma NEFA^1^ (mmol/L; antilog)	-	0.40	-	0.50	-	0.35	-	-
Plasma BHB^2^ (log10)	104	0.05	0.049	0.09	0.050	-0.05	0.047	0.12
Plasma BHB^2^ (mmol/L; antilog)	-	1.12	-	1.23	-	0.89	-	-
Milk C18:1 cis 9 wk 1–14 (% of milk)	220	0.93^ab^	0.035	0.99^a^	0.034	0.82^b^	0.032	0.003
ΔBCS^3^ wk 14—wk 1 (scale 1–5)	36	-0.59^b^	0.100	-0.53^b^	0.111	-0.24^a^	0.105	0.04
ΔBW^4^ wk 14—wk 1(kg)	36	-37.3	12.62	-48.0	13.98	-10.7	13.16	0.10

^1^Non-esterified fatty acids.

^2^β-hydroxybutyrate.

^3^Body condition score.

^4^Body weight.

^a–b^Means within rows with different superscripts differ significantly (P < 0.05).

The effects of RFI_NorFor_ group (LRFI_NorFor_, IRFI_NorFor_ or HRFI_NorFor_) during the whole 42-wk lactation are presented in Table 4. During the whole lactation, HRFI_NorFor_ cows yielded less ECM and was less efficient (ECM yield/kg DMI) than MRFI_NorFor_ and LRFI_NorFor_ cows. There was a breed effect on both DMI and ECM yield, with SH cows having higher DMI and yielding more ECM than SR cows. Neither DMI nor dry matter apparent digestibility were affected by RFI group. Throughout the lactation, BW was lower in LRFI_NorFor_ compared with IRFI_NorFor_ and HRFI_NorFor_ while BCS were higher in HRFI_NorFor_ cows than in LRFI_NorFor_ cows. The HRFI_NorFor_ cows had a positive weekly change in BCS during the 42-wk lactation period while it was negative for the other two RFI_NorFor_ groups. The persistency of lactation was lower in HRFI_NorFor_ than in IRFI_NorFor_ and LRFI_NorFor_ cows. Emissions of CH_4_/day, CH_4_/DMI and of CH_4_/ECM were all higher among HRFI_NorFor_ cows than IRFI_NorFor_ and LRFI_NorFor_ cows.

**Table 4:** Full-lactation performance (least square mean and SEM) of cows divided into two groups, with low residual feed intake (LRFI; n = 12), intermediate (IRFI; n = 12) or high RFI (HRFI; n = 12) according the NorFor (2011) equation (for parameter abbreviations, see main text).

*NorFor*								
Variable:	Obs.	LRFI	SEM	IRFI	SEM	HRFI	SEM	P-value
RFI_NorFor_ (MJ NE/d)	661	-5.55^c^	1.26	0.05^b^	1.46	8.89^a^	1.38	<0.001
DMI (kg/d)	686	24.3	0.31	25.1	0.35	25.0	0.33	0.12
ECM (kg/d)	686	37.0^a^	0.63	37.0^a^	0.72	32.9^b^	0.69	<0.001
DMD (%)	71	69.3	0.69	71.5	0.79	69.1	0.76	0.07
Efficiency (ECM/kg DMI)	686	1.54^a^	0.021	1.49^a^	0.024	1.32^b^	0.023	<0.001
BCS (scale 1–5)	1523	3.10^a^	0.063	3.27^ab^	0.072	3.44^b^	0.070	0.003
BCS weekly change (scale 1–5; BCS/week)	1474	-0.008^b^	0.0020	-0.004^ab^	0.0023	0.004^a^	0.0023	0.001
BW (kg)	1494	716^a^	9.9	755^b^	11.3	759^b^	10.7	0.006
BW weekly change (kg/week)	1457	0.77	0.636	1.24	0.682	2.73	0.697	0.12
Persistency (kg ECM wk 31 to 40/kg ECM wk 1 to 10)	36	0.80	0.045	0.78	0.055	0.64	0.051	0.05
CH_4_ (g/kg ECM)	1002	11.0^b^	0.35	10.9^b^	0.39	13.9^a^	0.38	<0.001
CH_4_ (g/d)	1023	402^b^	6.1	402^b^	6.9	426^a^	6.7	0.02
CH_4_ (g/kg DMI)	1023	16.7^b^	0.28	16.3^b^	0.32	17.5^a^	0.31	0.03

^a–c^Means within rows with different superscripts differ significantly (P < 0.05).

The effects of RFI_NRC_ (LRFI_NRC_, IRFI_NRC_ or HRFI_NRC_) during the whole 42-wk lactation are presented in Table 5. The LRFI_NRC_ cows consumed less feed than the IRFI_NRC_ and HRFI_NRC_ cows. There was a breed effect on both DMI and ECM yield, with SH cows having higher DMI and yielding more ECM than SR cows. Both ECM and dry matter apparent digestibility was not affected by RFI group. Efficiency, expressed as ECM yield/kg DMI, was lower in HRFI_NRC_ cows than in IRFI_NRC_ and LRFI_NRC_ cows. Both BW and BCS were not affected by RFI_NRC_ group. The persistency of lactation and emissions of CH_4_ and CH_4_/ECM were also not affected by RFI_NRC_ group_._ However, CH4/DMI was higher in LRFI_NRC_ compared with the other two groups.

**Table 5:** Full-lactation performance (mean and SEM) of cows divided into two groups, with low residual feed intake (LRFI) (n = 18) or high RFI (HRFI) (n = 18) according the NRC (2001) equation (for parameter abbreviations, see main text).

*NRC*								
Variable:	Obs.	LRFI	SEM	IRFI	SEM	HRFI	SEM	P-value
RFI_NRC_ (kg DM/d)	686	-2.77^c^	0.216	-1.54^b^	0.218	-0.37^a^	0.207	<0.001
DMI (kg/d)	686	23.7^a^	0.30	24.9^b^	0.31	25.7^b^	0.29	<0.001
ECM (kg/d)	686	35.5	0.77	36.5	0.78	34.8	0.74	0.30
DMD (%)	71	70.5	0.78	69.9	0.79	69.2	0.76	0.52
Efficiency (ECM/kg DMI)	686	1.52^a^	0.025	1.49^a^	0.026	1.36^b^	0.024	<0.001
BCS (scale 1–5)	1523	3.33	0.074	3.15	0.074	3.33	0.069	0.14
BCS weekly change (scale 1–5; BCS/week)	1474	-0.005^ab^	0.0022	-0.006^b^	0.0022	0.002^a^	0.0021	0.03
BW (kg)	1494	758	11.8	726	11.8	742	11.2	0.15
BW weekly change (kg/week)	1457	1.76	0.667	0.67	0.624	2.31	0.639	0.16
Persistency (kg ECM wk 31 to 40/kg ECM wk 1 to 10)	36	0.73	0.051	0.78	0.056	0.73	0.053	0.67
CH_4_ (g/kg ECM)	1002	12.0	0.47	11.5	0.47	12.5	0.45	0.31
CH_4_ (g/d)	1023	412	6.5	404	6.5	414	6.3	0.51
CH_4_ (g/kg DMI)	1023	17.6^a^	0.30	16.6^b^	0.30	16.5^b^	0.29	0.008

^a–c^Means within rows with different superscripts differ significantly (P < 0.05).

Pearson correlation between RFI_NorForFull-lact_ and RFI_NorFor_ estimates based on shorter test periods ranged from 0.54 to 0.73 for 14-wk periods in early, mid-, and late lactation. The correlation between the shorter test periods ranged from 0.01 to 0.26 (Table 6). Pearson correlation between RFI_NRCFull-lact_ and RFI_NRC_ estimates based on shorter test periods ranged from 0.68 to 0.79 for 14-wk periods in early, mid-, and late lactation. The correlation between the shorter test periods ranged from 0.2 to 0.4 (Table 7).

**Table 6:** Pearson correlation matrix for residual feed intake calculated using the NorFor (2011) equation (RFI_NorFor_), estimated using data collected at different stages of the lactation and for the full lactation (n = 36).

	RFI_NorFor Full-lact_	RFI_NorFor Early-lact_	RFI_NorFor Mid-lact_
RFI_NRC Early-lact_	0.538		
RFI_NorFor Mid-lact_	0.709	0.207	
RFI_NorFor Late-lact_	0.727	0.007	0.264

**Table 7:** Pearson correlation matrix for residual feed intake calculated using the NRC (2001) equation (RFI_NRC_), estimated using data collected at different stages of the lactation and for the full lactation (n = 36).

	RFI_NRC Full-lact_	RFI_NRC Early-lact_	RFI_NorFor Mid-lact_
RFI_NorFor Early-lact_	0.723		
RFI_NorFor Mid-lact_	0.677	0.204	
RFI_NorFor Late-lact_	0.787	0.303	0.401

### Discussion

An animal’s RFI value provides an estimate of its efficiency relative to the average animal in the cohort based on variables included in the model and their associated measurement error, plus any errors in fitting the model itself [2, 7]. In the present study, RFI over a complete lactation was calculated according to NRC [15] and according to NorFor [14]. Thirty-six multiparous cows were divided into three groups of equal size with low, intermediate or high RFI, respectively. Residual feed intake according to the NorFor equation did not affect DMI. However, the daily ECM yield was about 4 kg lower in the HRFI cows compared with the other two RFI groups. Cows with high RFI were, as expected, less efficient in terms of ECM/kg DM. In contrast, RFI according to the NRC equation was related to feed intake. Feed efficient LRFI_NRC_ cows showed lower DMI while ECM yield did not relate to RFI_NRC_ group. The result agrees with most previous studies indicating that low RFI according to NRC is a consequence of lower intake while ECM yield is maintained [2, 25]. The process of digestion may explain part of the variance in RFI [26, 27]. However, in the present study, apparent DMD did not differ between RFI groups. The DMD determinations were based on fecal spot samples. This method is less reliable compared with total collection and the results should thus be interpreted with caution. Cows in the HRFI_NorFor_ group showed higher daily CH_4_ production, CH_4_/ECM and CH_4_ yield [g/kg DMI] than MRFI_NorFor_ and LRFI_NorFor_ cows. Most studies agree that CH_4_/ECM generally is negatively related to milk production and that DMI is the main driver of daily CH_4_ output [23, 28]. The DMI intake did not differ between the three RFI_NorFor_ groups and it is thus possible that the higher emission in the HRFI_NorFor_ group reflects changes in the rumen microbial community [24]. Such differences in in the rumen microbial community among cows are linked with differences in the degree of CH_4_ production [24]. The result is intriguing since previous studies indicate that RFI was not related to CH_4_ yield and there was no overall effect on the methanogen community [29, 30]. In agreement with [31], RFI status according to the NRC equation did not affect daily CH_4_ production and CH_4_/ECM while CH_4_ yield increased in LRFI cows. The persistency of lactation tended (p = 0.05) to be influenced by RFI_NorFor_ status. Cows categorized as HRFI_NorFor_ showed a lower persistency than the two groups of cows categorized as more efficient. Capuco et al. [32] postulated that efficiency could be increased by improving persistency after reaching peak milk yield. It is possible that the tendency towards decreased persistency contributed to the reduced efficiency of the HRFI_NorFor_ cows in this study. On the other hand, persistency of the lactation curve was not affected in groups of cows with different RFI_NRC_ status_._ The LRFI_NorFor_ and IRFI_NorFor_ cows lost BCS over the lactation while HRFI_NorFor_ gained BCS. Also during the first 14 weeks of lactation, a similar pattern was observed with a more marked loss of BCS in LRFI_NorFor_ and IRFI_NorFor_ cows than in HRFI_NorFor_ cows. We chose to study variables related to energy balance during the first 14 weeks after parturition because many cows require that period to reach energy balance [16]. The average level of insulin in plasma was lower while NEFA was higher in LRFI_NorFor_ cows during the first six weeks of lactation. Insulin and NEFA reflects EB as reviewed by Leduc et al. [33]. A limitation with the present study is that blood was collected only at three occasions; a more frequent sampling might have given a more detailed picture of the metabolism. Never the less, a similar metabolic pattern was observed in cows categorized as HRFI according to NRC with a less pronounced loss of BCS both in early lactation and during the full lactation. Also, concentration of NEFA in plasma and the concentration of the fatty acid C18:1 *cis* 9 in milk was lower in HRFI_NRC_ cows. The latter fatty acid has recently been shown to be related to EB in early lactating cows [34] since it largely derives from mobilized adipose. Taken together, the results indicate that more efficient cows with lower residual feed intake, both according to NorFor and NRC, mobilized more of their body reserves both in early lactation and during the complete lactation until dry off. The result indicates that the high efficiency of LRFI cows partly was an artefact related to use of body reserves as previously suggested [7]. It appears as the NorFor equation underestimated the energy stored in the body reserves. Never the less, the net contribution of body reserves over the lactation could only explain a limited part of the differences in efficiency between the RFI groups. The cows, which were feed efficient according to the Norfor equation emitted less CH_4_ which, in turn, reduced energy losses related to rumen fermentation. The persistent shape of the lactation curve may also have contributed to the feed efficiency as mentioned above. However, based on a meta-analysis of 31 respiration chamber experiments, Guinguina et al. [35] concluded previously that as much as 65% of the variation in RFI between cows is explained by metabolic efficiency not related to digestion. It is reasonable to assume that also in the present study metabolic efficiency was significantly related to RFI_NorFor._ We assume that metabolic efficiency was the main factor contributing to the variation in RFI between efficiency groups also according to NRC. McNamara [36] reported that basic metabolic functions, directly related to metabolic efficiency, could vary by 20% among cows producing similar amounts of milk. It is important to note that RFI is only part of feed efficiency. Selection for efficiency must also consider the optimal levels of milk production relative to BW. In the present study the LRFI_NorFor_ cows were less heavy compared with their HRFI_NorFor_ counterparts. Body weight is actually genetically correlated negatively to feed efficiency [37].

Efficiency measurements across full lactations are costly and time-consuming, and therefore shorter test periods are normally used. However, in early lactation cows generally mobilize body resources and lose BW in order to maintain milk production, whereas cows in later stages of lactation accrete body resources. Thus, RFI measured during shorter periods than full lactations might be misleading. However, it has been shown that a test period of 64–70 d in duration between approximately 150 and 220 DIM can provide a strong approximation of RFI for a full lactation [6]. Nevertheless, it must be underlined that such correlations between sub-periods and the full lactation include a significant number of common observations, improving the relationship. In the present study, we divided the full lactation into three sub-periods of equal length and found that RFI measured during late lactation showed the strongest relationship with full-lactation RFI. The relationships between RFI in the three sub-periods were weak for both models, in line with previous results [5].

## Conclusions

Multiparous dairy cows characterized by high efficiency (both LRFI_NorFor_ and LRFI_NRC_ cows) over a complete lactation mobilized more of their body reserves both in early lactation and during the complete lactation until dry off. Highly efficient cows according to the NorFor model had lower BW and a tended to have a more persistent shape of the lactation curve than cows characterized as less efficient. More efficient cows according NorFor showed lower daily CH_4_ production, CH_4_/ECM and CH_4_ yield than low efficient cows. RFI status according to the NRC equation did not affect daily CH_4_ production and CH_4_/ECM while CH_4_ yield increased in efficient cows. The results also indicated great phenotypic variation in RFI between sub-periods of the lactation, and thus efficiency studies covering the complete lactation are recommended.

## Supporting information

S1 Data (XLSX)
